# Bedform segregation and locking increase storage of natural and synthetic particles in rivers

**DOI:** 10.1038/s41467-021-27554-4

**Published:** 2021-12-16

**Authors:** J. Dallmann, C. B. Phillips, Y. Teitelbaum, Edwin Y. Saavedra Cifuentes, N. Sund, R. Schumer, S. Arnon, A. I. Packman

**Affiliations:** 1 Department Mechanical Engineering, Northwestern University, Evanston, IL USA; 2 Center for Preparatory Studies, Nazarbayev University, Nur-Sultan, Kazakhstan; 3 Department of Civil and Environmental Engineering, Northwestern University, Evanston, IL USA; 4 Department of Civil and Environmental Engineering, Utah State University, Logan, UT USA; 5 Zuckerberg Institute for Water Research, Ben‐Gurion University of the Negev, Beersheba, Israel; 6 Desert Research Institute, Reno, NV USA

**Keywords:** Hydrology, Environmental impact

## Abstract

While the ecological significance of hyporheic exchange and fine particle transport in rivers is well established, these processes are generally considered irrelevant to riverbed morphodynamics. We show that coupling between hyporheic exchange, suspended sediment deposition, and sand bedform motion strongly modulates morphodynamics and sorts bed sediments. Hyporheic exchange focuses fine-particle deposition within and below mobile bedforms, which suppresses bed mobility. However, deposited fines are also remobilized by bedform motion, providing a mechanism for segregating coarse and fine particles in the bed. Surprisingly, two distinct end states emerge from the competing interplay of bed stabilization and remobilization: a locked state in which fine particle deposition completely stabilizes the bed, and a dynamic equilibrium in which frequent remobilization sorts the bed and restores mobility. These findings demonstrate the significance of hyporheic exchange to riverbed morphodynamics and clarify how dynamic interactions between coarse and fine particles produce sedimentary patterns commonly found in rivers.

## Abstract (web summary)

Here the authors show that hyporheic flow, bed morphology, and bed stability are intimately related, and that this relationship is expressed as distinct locked and segregated states of bedform dynamics, which carries implications for river system behavior in general and the storage of carbon, nutrients, and contaminants in particular.

## Introduction

Rivers carry both dissolved and particulate material from the continents to the oceans. Terrestrial particulate matter plays a key role in structuring alluvial river channels^1^, maintaining deltaic coastlines^2^, and supporting aquatic ecosystems^3,4^. Particulate organic matter is retained in riverbeds and floodplains^5^, buried in deltaic clinoforms^6^ and stored in marine sediments^7,8^. Consequently, internal river system dynamics regulate the metabolism of carbon, yielding an annual efflux of 5.1 Pg of carbon from rivers to the atmosphere, and delivering 0.9 Pg^9^ of terrestrially derived carbon to the oceans^5,8,10–12^. Land development and agriculture have substantially increased soil erosion and delivery of particulate matter to rivers^13^. Excessive accumulation of these fine particles in sediments (siltation, embeddedness) is one of the major causes of impairment of aquatic ecosystems today^14,15^. These impacts are greatly exacerbated when the particles are themselves toxic (e.g., metal mine tailings)^16^. Concurrently, large quantities of plastics have been introduced into aquatic systems, yielding extraordinary numbers of small particles, fragments, and fibers—collectively termed microplastics—that are transported through and accumulate within fluvial systems^17,18^. The storage times of such synthetic particles and their long-term consequences for aquatic ecosystems are currently unknown.

Terrestrial, aquatic, and anthropogenic particles are subject to a wide range of conditions during transport from river headwaters to coastal ecosystems, including sunlight and oxygen variations in the water column, physical abrasion, strong redox gradients, and diverse microbial metabolism in the riverbed^19–21^. Dissolved and particulate organic matter is transformed both in the stream and within the hyporheic zone—the highly bioactive region of the riverbed where river water mixes with groundwater^19^. Hyporheic exchange facilitates microbial metabolism by delivering oxygen, carbon, and nutrients to benthic and hyporheic microbial communities^19^. The rate and extent of hyporheic exchange are controlled by river flow, channel morphology, and riverbed permeability. Nevertheless, hyporheic flux and storage timescales have not been incorporated into numerical and conceptual models for the dynamics of particulate organic matter or microplastics in rivers^22–25^.

To date, deposition of fine (diameter < 50 $\mu {{{{{\rm{m}}}}}}$) and light (specific gravity ~1) inorganic, organic, and synthetic particles in riverbeds has not been considered because it is generally assumed that they remain suspended in the water column due to low settling velocities^26^. Although early studies indicated that particles that are fine and/or light may impact bed morphodynamics^27^ and fines are known to modulate fluid properties^28^, they are commonly assumed to only interact minimally with riverbeds^29^. Increasingly, there is awareness that fine particles can impact bed morphodynamics, as recent studies have shown that fines can change bed slope^30^ and interact with bed sediments as part of the bedload^31,32^. Moreover, fine suspended particles are transported into riverbeds by hyporheic exchange and accumulate in the subsurface^33–36^.

Here we show that fine-particle dynamics, hyporheic exchange and riverbed morphodynamics are highly coupled, and this coupling drives the system to one of two asymptotic end states: bedform locking in which fine particles accumulate within bedforms and completely stabilize the bed, and segregation in which fine particles propagate downward through bedforms, completely restoring bed morphodynamics and forming buried depositional layers. Both end states leave a distinctive depositional pattern that can be detected via sediment cores. Further, these end states control both particle retention timescales and bed remobilization frequencies, which regulate both the breakdown and ecological impact of fine particles in rivers.

## Results

### Fine suspended particles deposit in the bed and alter bed morphodynamics

**Fig. 1:**
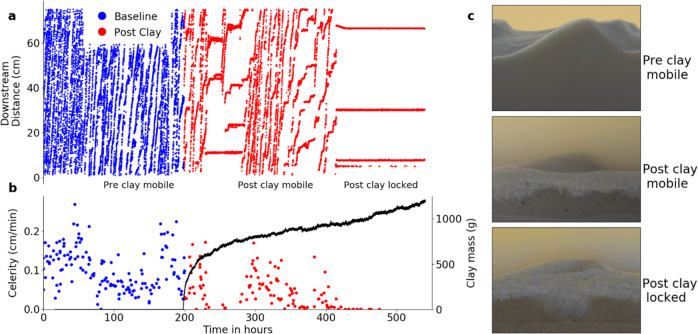
Temporal evolution of bedforms towards a locked end state. **a** Bedform troughs were continuously tracked both before (blue) and after clay additions (red). After the addition of clay, bedforms slow and eventually lock in place. Red horizontal lines indicate immobile bedforms (i.e., celerity = 0). **b** Red and blue points represent bedform celerities calculated for the trough locations shown in **a**, while the solid black line represents the accumulation of clay within the bed. As the clay accumulates, the bed temporally locks (celerities approach zero near 250 h) and then bed movement restarts due to upstream turbulent fluctuations. The bed relocks after sufficient clay accumulates in the bed (near 410 h). **c** From top to bottom, images showing clean bed mobile bedforms (50 h), post clay addition partially mobile bedforms (300 h), and locked bedforms (450 h), respectively. Images have been color matched to aid in visualization of the clay layer. Under conditions of high bed sediment transport rates, ongoing sand transport leads to a segregated end state with mobile bedforms propagating over a layer of deposited clay. However, in cases dominated by stabilization, extensive clay deposition within bedforms produces a locked end state.

**Fig. 2:**
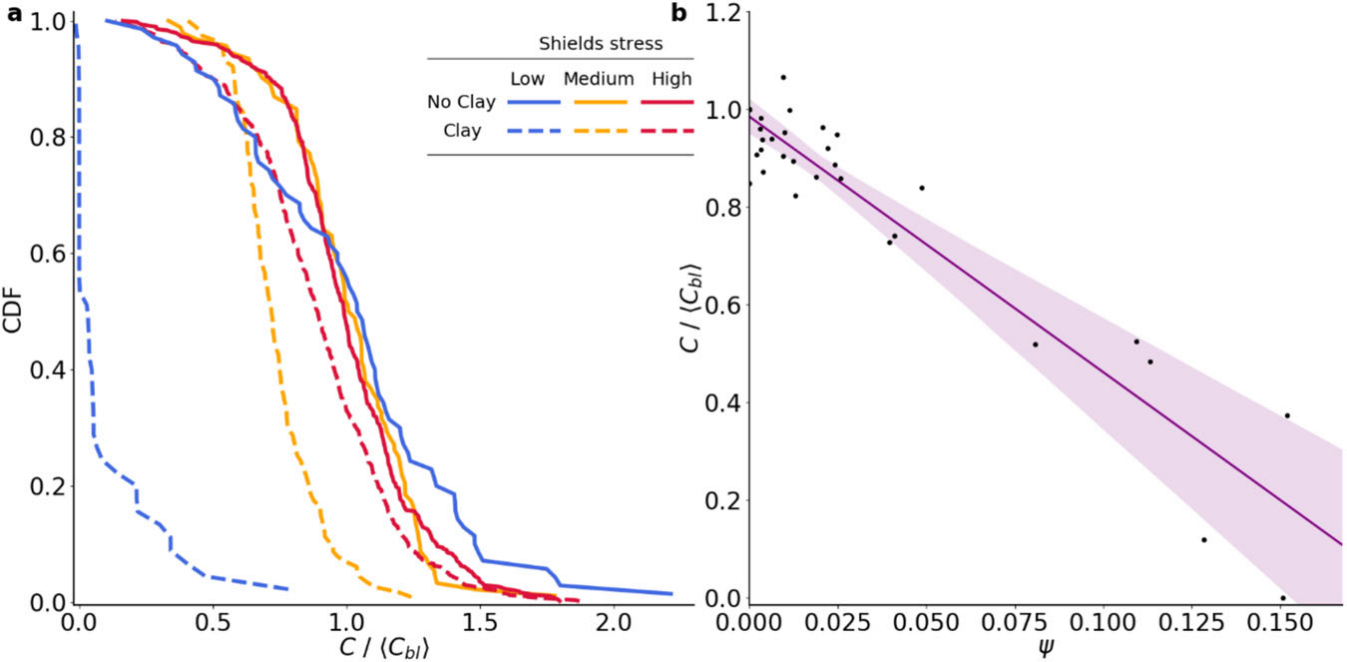
Interplay between Shields stress, clay deposition, and bedform morphodynamics. **a** Three examples of distributions of bedform celerity prior to (solid) and following (dashed) clay addition for low (blue), medium (yellow), and high (red) Shields stresses. The bedform celerity distribution decreases following clay deposition, and the reduction is more pronounced under smaller imposed fluid shear (blue lines). **b** Normalized bedform celerity decreases with the bedform stabilization ratio $\psi =\frac{M}{{\tau }_{* }}$, indicating that the observed morphodynamic changes reflect a balance between cohesive forces and the fluid Shields stress. Points represent the average of the 100 h of bedform celerity data. Linear fit (R^2^ = 0.93) with 95% confidence interval is shown. This relationship is consistent for both locking (stabilization dominated) and segregating (mobilization dominated) conditions.

We simultaneously observed bed morphodynamics and deposition of fine particles (kaolinite clay) in recirculating laboratory flumes under conditions typical of small sand-bed streams (Methods). We first observed the morphodynamics of sand alone (before adding clay) to assess distributions of bedform celerity and morphology under each flow condition (Fig. 1a, b). We then added dispersed clay to the freestream and observed streamflow, clay deposition, and changes in bed morphodynamics using a combination of imagery (bedforms and clay), acoustic Doppler velocimetry (flow and bedform statistics at-a-point), suspended clay concentration measurements (real time) and bed clay content (at the conclusion of the experiments). Clay was transported into and through the bed along hyporheic flow paths and deposited at the location of maximum hyporheic influx to each bedform and within bedform troughs (Supplementary Fig. 1, Supplementary Movie I). Clay accumulation stabilized the bed, reducing the bedform celerity (Fig. 1a, b), and altering bed morphology (Fig. 1c). This type of stabilization has been observed in granular mixtures^37,38^ and is known to result from particle–particle interactions (cohesion) as clay deposits fill pores and form bridges between sand grains^39–43^. We were not able to observe these microscale processes directly in our large-scale experiments, but clay deposition patterns, reduced bedform celerity, and altered bedform morphology all demonstrate the effects of stabilization (Fig. 1, Fig. 2, Supplementary Fig. 2).

In experiments with relatively low-shear velocities (U_*_ = 0.013 m/s), clay accumulation completely stabilized the bed, locking bedforms in place (Supplementary Movie I). Locking occurred when cohesion increased to such an extent that the imposed fluid shear was no longer sufficient to mobilize the bed. Bedform celerity decreased as clay accumulated in the bed (Fig. 1, Fig. 2), and exhibited stochastic behavior as the transport rate neared zero. Complete locking was preceded by periods of incipient locking, in which the bed became fully stabilized locally at the observation location but remained mobile upstream. The resultant sediment transport propagated through the system and episodically remobilized the bed in the measurement area (Fig. 1a–c). As the bed approached a locked state, partial stabilization substantially changed the bed morphology. Bedform wavelengths increased when bed sediment transport slowed (Supplementary Fig. 2). Further, during the period of bedform locking, a small amount of sand remained in transport over the locked bedforms. This combination of sand deposition in bedform troughs and overall lengthening of bedforms reduced bedform lee angles (Supplementary Fig. 2). Clay deposition ultimately locked the bed completely, halting sediment transport everywhere in the system. Fully locked bedforms had visibly different shapes and morphological properties than either clay-free or sorted clay-sand bedforms, as they are elongated and their lee angle is reduced noticeably. (Fig. 1c, Supplementary Fig. 2).

The extent of stabilization of the bed depended on the imposed fluid shear as well as the amount of deposited clay (Fig. 2). While clay floc size increased slightly with mass of clay used in each injection and salinity, floc size did not noticeably impact bed stabilization. Bedform celerity decreased in all cases, but complete locking only occurred under relatively low-shear conditions in which stabilization dominated the return of clay to the water column via hyporheic exchange and bedform scour (remobilization) (Fig. 2a). We assessed the change in bedform dynamics in terms of a stabilization ratio $\psi$, defined as the ratio of the clay fraction in the bed (M) (a proxy for the cohesive force associated with clay deposition) and the mobilization force imposed by the fluid (nondimensional Shields stress, ${\tau }_{* }$). The normalized bedform celerity (ratio of the mean celerity of clay-sand bedforms <C> relative to sand alone <C_b_>) decreased linearly with the stabilization ratio (Fig. 2b). Stabilization of mobile sediment beds solely by deposition of fine particles from the water column has not previously been quantified. These findings indicate that fine-particle deposition and remobilization episodically regulate the morphodynamics of sand-bed rivers.

## Discussion

### Clay-sand bed end states: competition between segregation and locking

**Fig. 3:**
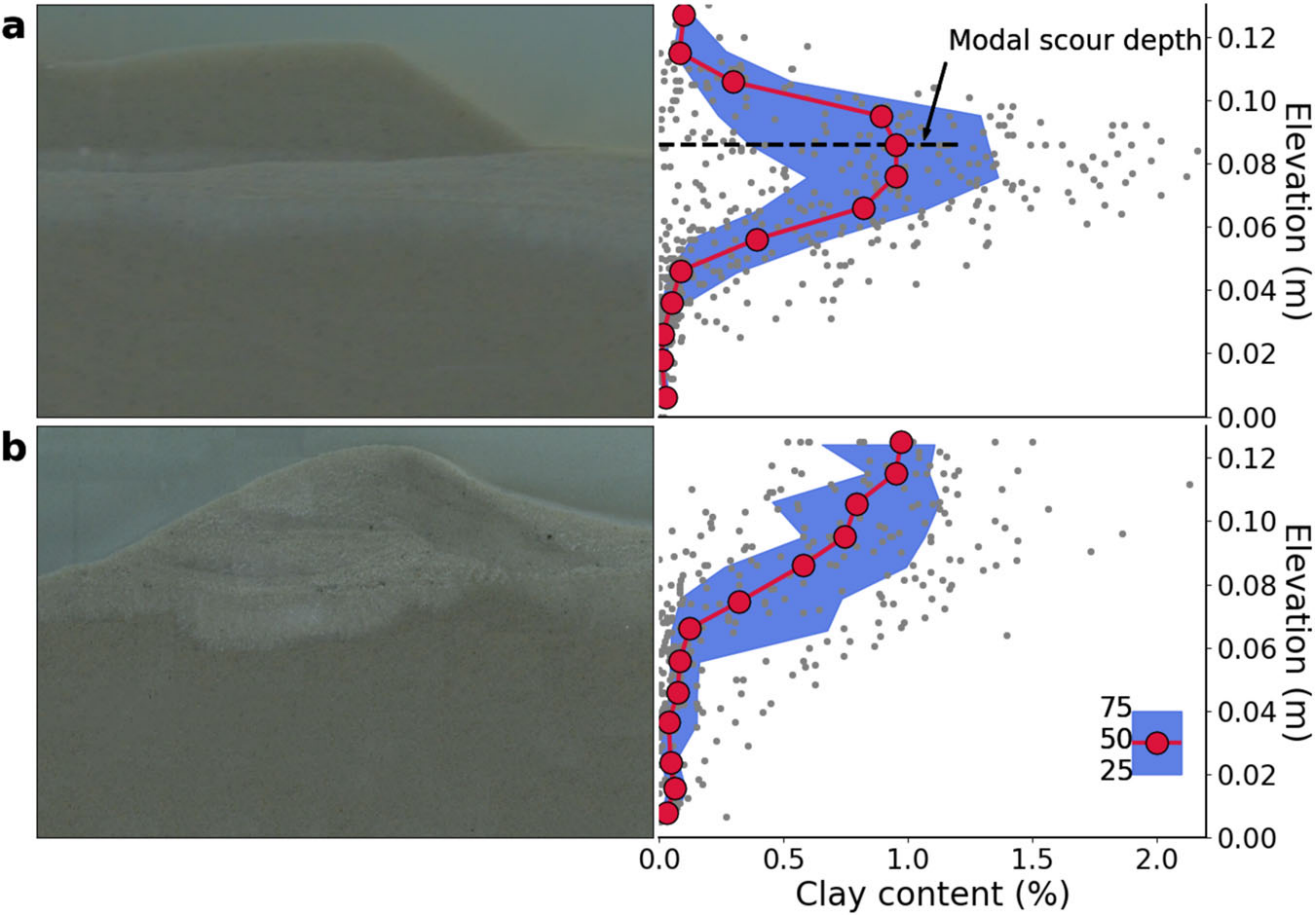
Clay accumulation patterns for segregated and locked end states. Images on the left are representative images of the two end states, while concentration profiles on the right reflect clay accumulation in the sediment bed (see methods). Red circles denote the median concentration and shading represents the interquartile range. **a** In the segregated end state (NU-3), clay accumulates in a defined layer just below the active layer of sand transport. The peak of the clay concentration profile occurs just below the most frequent bedform scour depth. Clay also deposits below the maximum scour depth (~0.075 m), as clay is actively pumped underneath bedforms by hyporheic exchange. **b** In the locked end state (NU-8), extensive clay accumulation within bedforms halts bed motion. The clay deposition patterns reflect bedform-induced hyporheic pumping into the stoss slope and through the bedform. In this case, there is no defined layer of buried clay and deposited clay concentration decreases monotonically with depth. Flow is from left to right in the images of bedforms. Images have been color matched to allow for easier visualization of clay accumulation.

Under conditions of high bed mobility ($\psi$ - > 0), deposited clay is frequently remobilized from within bedforms, and long-term deposition only occurs in a horizontal layer below the active region of bed sediment transport. For this case, we observed a peak in clay accumulation at the location of the most frequent (modal) scour depth (Fig. 3a). This can be considered the result of a stochastic process in which passage of a random series of bedforms induces both downward motion of suspended particles along hyporheic flow paths and remobilization of deposited particles though scour. This remobilization can be considered a type of winnowing process removing fine particles from the sediment bed. However, repeated passage of bedforms moves clay particles deeper into the bed, and ultimately into regions from which they are not remobilized^44^. The resulting clay accumulation layer is horizontal because it is formed by the passage of many bedforms, which longitudinally homogenizes the effects of hyporheic exchange processes^45^. Conversely, when stabilization dominates, there is extensive deposition of clay within each bedform and the resulting strong local stabilization slows and ultimately stops bed sediment motion. For the locked case, we observed that clay accumulation decreased monotonically with depth in the bed (Fig. 3b), as expected for a process driven by flux of sediment particle from the water column^46,47^.

In the mobilization-dominated case, presence of clay in the mobile layer still decreased bedform celerity over the timescale of the experiment (Fig. 2), but clay did not permanently accumulate in this region. Instead, clay accumulated primarily below the active layer of sand transport, at depths where clay was delivered by hyporheic exchange but only infrequently remobilized by the passage of larger bedforms. Clay accumulation stabilized the bed at this depth, shifting the scour distribution upwards and reducing the mean bedform height (Supplementary Fig. 4). These dynamics produced a segregated end state in which clay accumulates just underneath the active layer, while maintaining a mobile layer of sand transport (Fig. 3a). Hyporheic exchange decreased by more than a factor of two at the depth of clay accumulation but was maintained within the active layer (Supplementary Fig. 1, Supplementary Movie 2).

The two morphodynamic end states observed here—segregated and locked—represent the asymptotic outcomes of stochastic forcing and internal dynamics in rivers. Bedforms develop spontaneously from interactions between river flow, bed sediment motion, and riverbed topography^48^. While suspended and bed particle dynamics were previously thought to be independent, our results show that hyporheic exchange and subsequent deposition of fine particles strongly modulate local bed morphodynamics. Over longer timescales, extensive repetition of these processes is expected to drive riverbeds to either the segregated or locked state. Highly mobile sand-bed rivers have little clay in the active layer while bed sediments in locked sand/silt/clay-bed rivers contain a mixture of coarse and fine particles^48^. The results presented here show that flow-bed-suspension dynamics reinforce these patterns. Further, while clay-sand sorting is normally assumed to be driven by wash-out of fine particles from mixed sediment beds, the results presented here show that fines are retained to a much greater extent than previously believed and accumulate in buried depositional layers. Such layered heterogeneity is known to occur in rivers and to strongly influence rates and patterns of hyporheic exchange^49^, but available field data do not resolve the scales of heterogeneity observed here^50,51^.

Larger-scale variations in flow and sediment inputs are expected to reinforce local bedform processes. Particles that are immobilized either by locking or by depositing below the active layer can only be remobilized under higher fluid shear, e.g., in floods. Floods generate larger bedforms with the capability to remobilize deposited fines from within stream channels^35^. However, floods also induce larger-scale hyporheic exchange processes and drive fine particles deeper into the streambed^34,52^. Therefore, both the mobilization and deposition processes observed here continue to occur during floods. The wide ranges of flow and morphodynamic conditions found in rivers are expected to increase the length and timescales of the processes we observed. Moreover, our observations support the recent hypothesis that fine sediment contributes to development of low-angle bedforms in large rivers, and provide an additional mechanism for development of unusual dune morphologies and sedimentary deposits^53^.

### Implications for storage and breakdown of natural and synthetic particulate matter

Both the locked and segregated end states have direct implications for fine-particle storage and metabolism in rivers. In the locked case, particles are trapped within bedforms until a high-flow event exceeds the bed erosion threshold. This increases particle residence times within the hyporheic zone to flood recurrence timescales. Fine-particle storage timescales are expected to be even greater in larger rivers, as these rivers require a sustained increase in discharge to modulate bedform morphodynamics, resulting in slow readjustment times^54^. In the segregated case, burial of fine particles beneath the active layer and the resulting limitation on hyporheic exchange both favor long-term retention of natural and synthetic particulate matter in rivers. Fine particles primarily deposit in a layer below the average scour depth and migrate further downward over time. Repeated flood events will drive this material deeper into the bed and form low-permeability strata underneath the river channel that restrict hyporheic exchange and decrease delivery of solutes from the overlying river. This process provides a mechanism for suspended particulate organic matter to be deposited, retained, and preserved under river channels.

Both end states increase the opportunity for metabolism of organic matter relative to current models that assume these particles remain in the water column. While particulate organic carbon is known to be buried and stored within floodplains^8^ and deltas^5,6^, our observations are the first to identify a clear mechanism for storage under active river channels.

This process likely contributes to the supersaturation of CO_2_ commonly found in rivers^55^ and the resulting high rates of outgassing to the atmosphere^10^.

Microplastics will similarly become buried and retained for long periods of time in riverbeds. Microplastics are colonized by biofilms^36^, and the sorption of ions and organic material to their surfaces leads to cohesive organic-inorganic aggregates that will contribute to bedform segregation. Over alluvial river valley morphodynamic timescales, channel migration leaves fluvial deposits buried within floodplains. The long-term structure formed by the processes observed here will be discontinuous and elongated fine-particle lenses, which will retain the signature of human development in the form of extensive fine-particle deposits containing large numbers of synthetic microplastic particles.

While the strength of bed cohesion will be modified by the size and surface properties of the suspended sediment and the porosity of the sand bed, the suspended flocculated clay diameter ($< 50\,\mu m$), suspended sediment concentrations ($< 10\,g/L$), and bed sediment diameter (${D}_{50}0.420\,{mm}$) used in this study are typical for many watercourses^56–58^. Both segregated and locked end states appear to occur frequently in natural systems. Riverbeds often contain surficial sand bedforms overlying subsurface fine-particle layers^49,59,60^. Field studies have indicated that the formation of these deposits can be connected to the interplay between hyporheic deposition and mobile bedform scour^34,35^. Clay in intertidal bedforms, where these layers are also present^44^, has been tied to decreases in bedform celerity^39^. Beds in these systems have high fractions of cohesive fine particles^61–64^ and are usually immobile^48^.

Our results show that complex feedbacks between fine-particle deposition, hyporheic exchange, and bedform morphodynamics increase the retention and burial of particles in rivers. The effects of bed segregation and locking processes need to be investigated in a variety of rivers to improve assessment of particle cycling between terrestrial, freshwater, and marine systems, re-evaluate the opportunity for metabolism of both terrestrially derived and aquatic organic matter in fluvial systems, and determine the long-term ecological impacts of synthetic particles. Riverine storage, siltation and metabolism of carbon, nutrients, and contaminants are expected to become more important in the future as increasing land development and precipitation intensity deliver more terrestrial particulate matter to rivers^65^. Our findings provide a basis for incorporating self-organized subsurface heterogeneity and coupled fine-coarse particle dynamics in models of riverine geomorphology, biogeochemistry, and ecosystem impacts.

## Methods

Sediment transport within sand-bed rivers and streams occurs at high Shields stresses (${{{{{{\rm{\tau }}}}}}}_{* }={{{{{\rm{\tau }}}}}}/\left({{{{{{\rm{\rho }}}}}}}_{{{{{{\rm{s}}}}}}}-{{{{{\rm{\rho }}}}}}\right){{{{{\rm{gD}}}}}}$) where flux occurs through the suspension of bed material and coherent bedform motion^41,42,53^. In this equation, τ is the shear stress (Pa), g is gravity (m/s^2^), D is a representative grain size (m), and ρ_s_ and ρ are the sediment and fluid density taken as 2650 and 1000 kg/m^3^, respectively. Bed morphodynamics depend primarily on freestream properties (e.g., velocity, depth), river reach geometry (e.g., slope, width), and the composition of the bed (grain size, roughness)^66,67^. Fine suspended particles (diameter < 50 $\mu {{{{{\rm{m}}}}}}$) are typically considered to not interact significantly with the bed based on an inferred low likelihood of deposition based on the particle settling velocity (${U}_{s}$) and hydrodynamic mixing, typically represented by the dimensionless Rouse number ($P=\frac{{U}_{s}}{\beta \kappa {U}^{* }}$). However, a recent reanalysis of suspended sediment profiles in sand-bed rivers suggests that suspended and bed sediments are in dynamic equilibrium^32^. Further, a growing body of evidence indicates that fine particles are transported into and accumulate within the bed due to hyporheic exchange^33–35^.

To explore the interactions between suspended particle dynamics and bed morphodynamics, we conducted experiments within two similar recirculating flumes at Northwestern University (NU)^40^ and Ben-Gurion University of the Negev (BGU) with mobile sediment beds and freestream kaolinite clay with a median listed particle diameter of 0.5 $\mu m$ and a flocculated diameter of $< 50\,\mu m$. Nine experiments were conducted at NU and three at BGU. All experiments were conducted with a constant freestream velocity but different background salinity, shear velocity, and the frequency and magnitude of clay injections (Supplementary Table 1). All experiments started with a flat bed composed entirely of sand with a D_50_ of 0.420 mm, which was allowed to fully develop prior to the addition of kaolinite. Shear velocity was was determined by fitting a log law velocity profile to a time-averaged downstream velocity profile over the fully developed bed. Sand-bed morphodynamics were observed for at least 70 h, which was the minimum time required for bedform statistics to converge. After the bed was fully developed and baseline morphological measurements were completed, suspended clay was added as either a single addition (7 runs) or in sequential additions (5 runs).

Bedform height (*H*), length (*L*) and celerity (*C*), and bed elevation were continuously measured both before and after clay injection. Bedform morphodynamics were measured using sidewall-mounted Nikon D5300 cameras. Images were processed using a simple black/white thresholding procedure (MATLAB R2019a) to extract the interface between the overlying fluid and the bedform. The peaks and troughs of each bedform were determined using a “find peaks” algorithm (Python 3.7 SciPy). Bedform length was calculated as the average distance between successive troughs, while celerity was determined via linear regression of the bedform trough displacement over time. A Nortek Acoustic Doppler Velocimeter (ADV) profiler was also used to continuously measure the bed elevation at single point. These data were processed with a Savitzky-Golay filter and a “find peaks” algorithm allowed for the extraction of the peaks and troughs. The troughs were used to generate the scour depth distribution for each run. Bedform height *H* was determined as the difference between the bedform crest and downstream (stoss side) trough.

The concentration of suspended clay in the freestream was measured continuously using Xylem turbidity meters (WTW Visoturb 700IQ SW for low concentrations and WTW Visolid 700IQ SW for high concentrations). Hyporheic exchange flux was measured periodically via salt tracer injections, with the in-stream salt concentration measured using a salinity meter (SM—Star Comm, resolution of 0.01 $\mu S/{cm}$). Hyporheic exchange was measured for the clean sand bed (before clay addition) and at various intervals throughout the experiment^47^. Clay concentration profiles in the bed sediment were obtained by taking cores at the conclusion of each run following methods of Dallmann 2020^40^. Once removed, the cores were sectioned, and the clay content of each section was measured by resuspending the deposited clay in DI water and then measuring light absorbance with a spectrometer (Hach Company, DR/4000). A calibration curve was used to relate sample absorbance to clay mass.

## Supplementary information

Supplementary Information

Description of Additional Supplementary Files

Supplementary Movie 1

Supplementary Movie 2

## Data Availability

Figures 1–3 and Supplementary Figs. 2–6 have associated raw data that are publicly available from the Hydroshare repository (https://www.hydroshare.org/resource/0983ec823ac64088b25882a6e99341ab/).

## Code availability

All codes used to process the raw data are available upon request. Inquiries should be addressed to Jon Dallmann at jonathandallmann2020@u.northwestern.edu.

## References

[1] Dunne KBJ, Jerolmack DJ (2018). Evidence of, and a proposed explanation for, bimodal transport states in alluvial rivers. Earth Surf. Dyn..

[2] Orton GJ, Reading HG (1993). Variability of deltaic processes in terms of sediment supply, with particular emphasis on grain size. Sedimentology.

[3] Likens GE, Bormann FH (1974). Linkages between terrestrial and aquatic ecosystems. BioScience.

[4] Withers PJ, Jarvie HP (2008). Delivery and cycling of phosphorus in rivers: a review. Sci. Total Environ..

[5] Battin TJ (2008). Biophysical controls on organic carbon fluxes in fluvial networks. Nat. Geosci..

[6] Leithold EL, Blair NE, Wegmann KW (2016). Source-to-sink sedimentary systems and global carbon burial: a river runs through it. Earth Sci. Rev..

[7] Masiello CA, Druffel ERM (1998). Black carbon in deep-Sea sediments. Science.

[8] Coppola AI (2018). Global-scale evidence for the refractory nature of riverine black carbon. Nat. Geosci..

[9] Drake TW, Raymond PA, Spencer RGM (2018). Terrestrial carbon inputs to inland waters: a current synthesis of estimates and uncertainty. Limnol. Oceanogr. Lett..

[10] Raymond PA (2013). Global carbon dioxide emissions from inland waters. Nature.

[11] Allen GH, Pavelsky TM (2018). Global extent of rivers and streams. Science.

[12] Galy V, France-Lanord C, Lartiges B (2008). Loading and fate of particulate organic carbon from the Himalaya to the Ganga–Brahmaputra delta. Geochim. Cosmochim. Acta.

[13] Borrelli P (2017). An assessment of the global impact of 21st century land use change on soil erosion. Nat. Commun..

[14] Hartwig M, Borchardt D (2015). Alteration of key hyporheic functions through biological and physical clogging along a nutrient and fine-sediment gradient. Ecohydrology.

[15] Merill L, Tonjes DJ (2014). A review of the hyporheic zone, stream restoration, and means to enhance denitrification. Crit. Rev. Environ. Sci. Technol..

[16] Feris K (2003). Differences in hyporheic-zone microbial community structure along a heavy-metal contamination gradient. Appl. Environ. Microbiol..

[17] Nel HA, Dalu T, Wasserman RJ (2018). Sinks and sources: assessing microplastic abundance in river sediment and deposit feeders in an Austral temperate urban river system. Sci. Total Environ..

[18] Frei, S. et al. Occurence of microplastics in the hyporheic zone of rivers. *Sci. Rep.*10.1038/s41598-019-51741-5 (2019). 10.1038/s41598-019-51741-5 PMC6813303 31649312

[19] Boano F (2014). Hyporheic flow and transport processes: mechanisms, models, and biogeochemical implications. Rev. Geophys..

[20] Battin TJ, Besemer K, Bengtsson MM, Romani AM, Packmann AI (2016). The ecology and biogeochemistry of stream biofilms. Nat. Rev. Microbiol..

[21] Canfield DE, Glazer AN, Falkowski PG (2010). The evolution and future of Earth’s nitrogen cycle. Science.

[22] Strååt KD, Mörth C-M, Sobek A, Smedberg E, Undeman E (2016). Modeling total particulate organic carbon (POC) flows in the Baltic Sea catchment. Biogeochemistry.

[23] Oeurng C, Sauvage S, Sánchez-Pérez J-M (2011). Assessment of hydrology, sediment and particulate organic carbon yield in a large agricultural catchment using the SWAT model. J. Hydrol..

[24] Schlesinger, W. H. & Bernhardt, E. *Biogeochemistry, An Analysis of Global Change*. 3rd edn, 688 (Academic Press, 2013).

[25] Bellasi, A. et al. Microplastic contamination in freshwater environments: a review, focusing on interactions with sediments and benthic organisms. *Environments*10.3390/environments7040030 (2020).

[26] Dawson, J. J. C. in *Ecosystem Services and Carbon Sequestration in the Biosphere* (eds. Rattan Lal et al.) 183–208 (Springer Netherlands, 2013).

[27] Simons, D. B., Richardson, E. V. & Haushild, W. L. Some effects of fine sediment on flow phenomena. In *Water Supply Paper 1498-G* (U.S. Geological Survey, 1963).

[28] Baas JH, Best JL (2008). The dynamics of turbulent, transitional and laminar clay-laden flow over a fixed current ripple. Sedimentology.

[29] Garcia, M. *Sedimentation engineering: Processes, measurements, modeling, and practice*. (American Society of Civil Engineers, 2008).

[30] Hill KM, Gaffney J, Baumgardner S, Wilcock P, Paola C (2017). Experimental study of the effect of grain sizes in a bimodal mixture on bed slope, bed texture, and the transition to washload. Water Resour. Res..

[31] Ma H (2017). The exceptional sediment load of fine-grained dispersal systems: example of the Yellow River, China. Sci. Adv..

[32] Lamb MP (2020). Mud in rivers transported as flocculated and suspended bed material. Nat. Geosci..

[33] Drummond JD, Aubeneau AF, Packman AI (2014). Stochastic modeling of fine particulate organic carbon dynamics in rivers. Water Resour. Res..

[34] Harvey, J. W. et al. Hydrogeomorphology of the hyporheic zone: stream solute and fine particle interactions with a dynamic streambed. *J. Geophys. Res. Biogeosci.*10.1029/2012JG002043 (2012).

[35] Phillips CB, Dallmann JD, Jerolmack DJ, Packman AI (2019). Fine‐particle deposition, retention, and resuspension within a sand‐bedded stream are determined by streambed morphodynamics. Water Resour. Res..

[36] Drummond, J. D., Nel, H. A., Packman, A. I. & Krause, S. Significance of hyporheic exchange for predicting microplastic fate in rivers. *Environ. Sci. Technol. Lett.*10.1021/acs.estlett.0c00595 (2020).

[37] Mandal S, Nicolas M, Pouliquen O (2020). Insights into the rheology of cohesive granular media. Proc. Natl Acad. Sci. USA.

[38] Baker ML (2017). The effect of clay type on the properties of cohesive sediment gravity flows and their deposits. J. Sediment. Res..

[39] Lichtman ID (2018). Bedform migration in a mixed sand and cohesive clay intertidal environment and implications for bed material transport predictions. Geomorphology.

[40] Dallmann, J. et al. Impacts of suspended clay particle deposition on sand‐bed morphodynamics. *Water Resour. Res.*10.1029/2019wr027010 (2020).

[41] Baas JH, Davies AG, Malarkey J (2013). Bedform development in mixed sand–mud: the contrasting role of cohesive forces in flow and bed. Geomorphology.

[42] Baas JH (2019). Integrating field and laboratory approaches for ripple development in mixed sand-clay-EPS. Sedimentology.

[43] Malarkey J (2015). The pervasive role of biological cohesion in bedform development. Nat. Commun..

[44] Wu X (2018). Wave ripple development on mixed clay‐sand substrates: effects of clay winnowing and armoring. J. Geophys. Res. Earth Surf..

[45] Packman AI, Brooks NH (2001). Hyporheic exchange of solutes and colloids with moving bed forms. Water Resour. Res..

[46] Preziosi‐Ribero, A. et al. Fine sediment deposition and filtration under losing and gaining flow conditions: a particle tracking model approach. *Water Resour. Res.*10.1029/2019wr026057 (2020).

[47] Fox A, Packman AI, Boano F, Phillips CB, Arnon S (2018). Interactions between suspended kaolinite deposition and hyporheic exchange flux under losing and gaining flow conditions. Geophys. Res. Lett..

[48] Wohl, E. in *Rivers in the Landscape* 125–195 (Wiley-Blackwell, 2020).

[49] Korus JT, Fraundorfer WP, Gilmore TE, Karnik K (2020). Transient streambed hydraulic conductivity in channel and bar environments, Loup River, Nebraska. Hydrol. Process..

[50] Salehin, M., Packman, A. I. & Paradis, M. Hyporheic exchange with heterogeneous streambeds: laboratory experiments and modeling. *Water Resour. Res.*10.1029/2003wr002567 (2004).

[51] Sawyer, A. H., Bayani Cardenas, M. & Buttles, J. Hyporheic temperature dynamics and heat exchange near channel-spanning logs. *Water Resour. Res.*10.1029/2011wr011200 (2012).

[52] Drummond JD (2014). Retention and remobilization dynamics of fine particles and microorganisms in pastoral streams. Water Res..

[53] Cisneros J (2020). Dunes in the world’s big rivers are characterized by low-angle lee-side slopes and a complex shape. Nat. Geosci..

[54] Martin RL, Jerolmack DJ (2013). Origin of hysteresis in bed form response to unsteady flows. Water Resour. Res..

[55] Butman D, Raymond PA (2011). Significant efflux of carbon dioxide from streams and rivers in the United States. Nat. Geosci..

[56] Fernández, R., García, M. H. & Parker, G. Upper Mississippi river flow and sediment characteristics and their effect on a harbor siltation case. *J. Hydraulic Eng.*10.1061/(asce)hy.1943-7900.0001507 (2018).

[57] Burrows, R. L., Emmett, W. W. & Parks, B. Sediment transport in the Tanana River near Fairbanks, Alaska, 1977–1979. *In Water-Resources Investigations Report 81-20* (U.S. Geological Survey, Reston, VA, 1981).

[58] Williams, C. A., Schaffrath, K. R., Elliott, J. G. & Richards, R. J. *Application of sediment characteristics and transport conditions to resource management in selected main-stem reaches of the Upper Colorado River, Colorado and Utah*. 10.3133/sir20125195 (2013).

[59] Flexser, S. *Lithologic Composition and Variability of the Sediments Underlying Kesterson Reservoir As Interpreted from Shallow Cores*. (Earth Sciences Division: Lawrence Berkeley Laboratory, 1988).

[60] Lu, C. et al. The influences of a clay lens on the hyporheic exchange in a sand dune. *Water*10.3390/w10070826 (2018).

[61] Healy, T., Wang, Y. & Healy, J.-A. *Muddy Coasts of the World: Processes, Deposition and Function*. (Proceedings in Marine Science, 2002).

[62] Schindler RJ (2015). Sticky stuff: redefining bedform prediction in modern and ancient environments. Geology.

[63] Amoudry, L. O. & Souza, A. J. Deterministic coastal morphological and sediment transport modeling: a review and discussion. *Rev. Geophys.*10.1029/2010rg000341 (2011).

[64] te Slaa S, He Q, van Maren DS, Winterwerp JC (2013). Sedimentation processes in silt-rich sediment systems. Ocean Dyn..

[65] Hayhoe, K. et al. *Impacts, Risks, and Adaptation in the United States: Fourth National Climate Assessment*, Volume II. 72–144 (U.S. Global Change Research Program, 2018).

[66] van Rijn LC (1984). Sediment transport, Part I: Bed load transport. J. Hydraulic Eng..

[67] Paarlberg, A. J., Dohmen-Janssen, C. M., Hulscher, S. J. M. H. & Termes, P. Modeling river dune evolution using a parameterization of flow separation. *J. Geophys. Res.*10.1029/2007jf000910 (2009).

