# Supplementary material for: Bedform segregation and locking increase storage of natural and synthetic particles in rivers
Source: Nat Commun. 2021 Dec 16;12:7315. doi: 10.1038/s41467-021-27554-4 (PMC8677759; doi:10.1038/s41467-021-27554-4)
Supplement: Supplementary file 1 — Supplementary Information [file 41467_2021_27554_MOESM1_ESM.pdf]

Supporting Information for

# Bedform segregation and locking increase storage of natural and synthetic particles in rivers

**Authors:** J. Dallmann[1,2], C.B. Phillips[3,4], Y. Teitelbaum[5], Edwin Y. Saavedra Cifuentes[3], N. Sund[6], R. Schumer[6], S. Arnon[5], A.I. Packman[3 *]

[1] Department Mechanical Engineering, Northwestern University, Evanston, IL, USA
[2] Center for Preparatory Studies, Nazarbayev University, Kazakhstan
[3] Department of Civil and Environmental Engineering, Northwestern University, Evanston, IL, USA
[4] Department of Civil and Environmental Engineering, Utah State University, Logan, UT, USA
[5] Zuckerberg Institute for Water Research, Ben-Gurion University of the Negev, Beersheba, Israel
[6] Desert Research Institute, Reno, Nevada, USA
[*]Corresponding Author

## Introduction

This supplementary information file contains two product datasheets, a table and six figures in support of the primary findings within the main text. The tables document the characteristics of the 12 experimental runs totaling nearly 5,000 hours of observations and the material used in the study. The figures support the main text by showing the results for all experiments.

***Supplementary Table 1: Details of the experimental conditions within each experiment.*** *Shear velocity was calculated using an acoustic Doppler velocimeter to determine velocity as a function of depth. The background salinity, number of additions and the size of each addition were selected to gain a variety of flow and fine particle loading conditions. NU denotes Northwestern University and BGU denotes Ben Gurion University of the Negev.*

| **Run ID** | **Shear Velocity (m/s)** | **Background Salinity (ppt)** | **Number of additions** | **Size of additions (g)** | **Total Added Mass (g)** | **Mean Baseline Height (m)** | **Mean Baseline Length (m)** | **Mean Baseline Celerity (m/hr)** |
|---|---|---|---|---|---|---|---|---|
| **NU-1** | 0.026 | 0.2 | 1 | 1000 | 1000 | 0.0227 | 0.777 | 0.770 |
| **NU-2** | 0.026 | 0.2 | 3 | 333 | 1000 | 0.0235 | 0.918 | 0.904 |
| **NU-3** | 0.026 | 0.2 | 17 | 300 | 5500 | 0.0234 | 0.865 | 0.870 |
| **NU-4** | 0.026 | 0.2 | 1 | 5500 | 5500 | 0.0223 | 0.838 | 1.039 |
| **NU-5** | 0.081 | 35 | 1 | 2000 | 2000 | 0.0227 | 0.877 | 2.367 |
| **NU-6** | 0.081 | 17.5 | 1 | 2000 | 2000 | 0.0232 | 0.877 | 2.554 |
| **NU-7** | 0.081 | 0.2 | 1 | 2000 | 2000 | 0.0233 | 0.877 | 2.234 |
| **NU-8** | 0.013 | 17.5 | 1 | 2000 | 2000 | 0.0138 | 0.227 | 0.062 |
| **NU-9** | 0.013 | 35 | 1 | 2000 | 2000 | 0.0138 | 0.227 | 0.062 |
| **BGU-1** | 0.013 | 0.2 | 4 | 320 | 1280 | 0.0165 | N/A | 0.088 |
| **BGU-2** | 0.013 | 0.2 | 5 | 200 | 1000 | 0.0165 | N/A | 0.088 |
| **BGU-3** | 0.013 | 0.2 | 8 | 80 | 640 | 0.0165 | N/A | 0.088 |

*Supplementary Table 2: Material used in the experiment.*

| Material | Particle $D_{50}$ (μm) | Particle $D_{50}$ (μm) | Main Chemical Components | Manufacturer | Part Number |
|---|---|---|---|---|---|
| **Kaolinite Clay** | 0.5 | 5 | $SiO_2$, $Al_2O_3$ | Unimin Specialty Materials | Snobrite75 |
| **Sand** | 420 | 600 | $SiO_2$ | US Silica | F-35 |

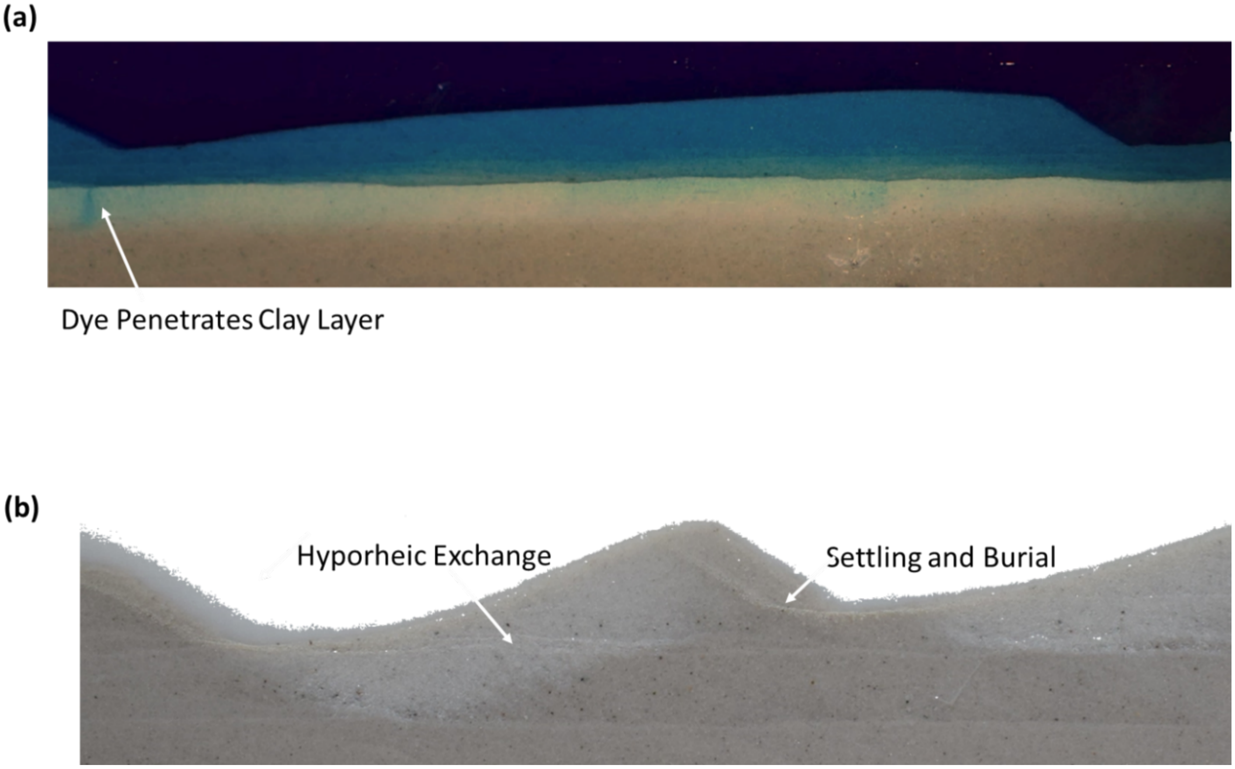


**Supplementary Figure 1:** a) Hyporheic exchange for Run NU-7 illustrated via dye injection into the freestream. This picture was taken 1.3 hours after the dye was added. The dye has filled the active layer but is blocked by the low permeability clay layer below the bedforms. However, smaller amounts of exchange still occur via localized penetration of the layer, as noted in the image. After ~24 hours dye permeates the entire subsurface. See Extended Data Video 2 for a time lapse video of this process. (b) Clay in the subsurface 25 minutes after the clay injection for Run NU-9. Clay deposition on the upstream side (left) is illustrative of the flow pattern created by hyporheic exchange. Clay settling in the troughs, where it is buried, is also visible. Hyporheic exchange rates were measured for NU-1,2 and 3 and the HEF was reduced by at least a factor of two (Dallmann et al., 2020).

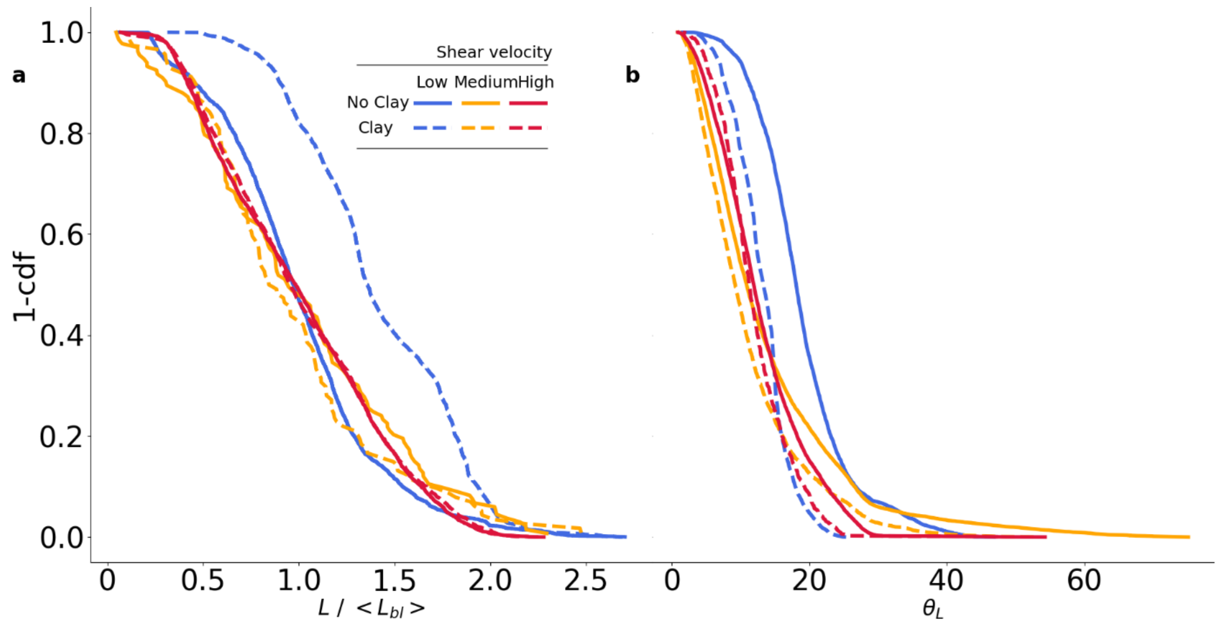


**Supplementary Figure 2:** (a) Distributions (1-cdf) of bedform length before and after clay was added for three sample runs at different shear velocities. The low, medium, and high shear velocity runs are NU-8, 4 and 6, respectively. Bedform length does not change for the medium and high shear velocity cases, but bedforms noticeably elongate for the low shear velocity case. (b) Distributions (1-cdf) of the downstream lee angle. The lee angle goes down when clay is added for all runs, most noticeably for the low shear velocity case.

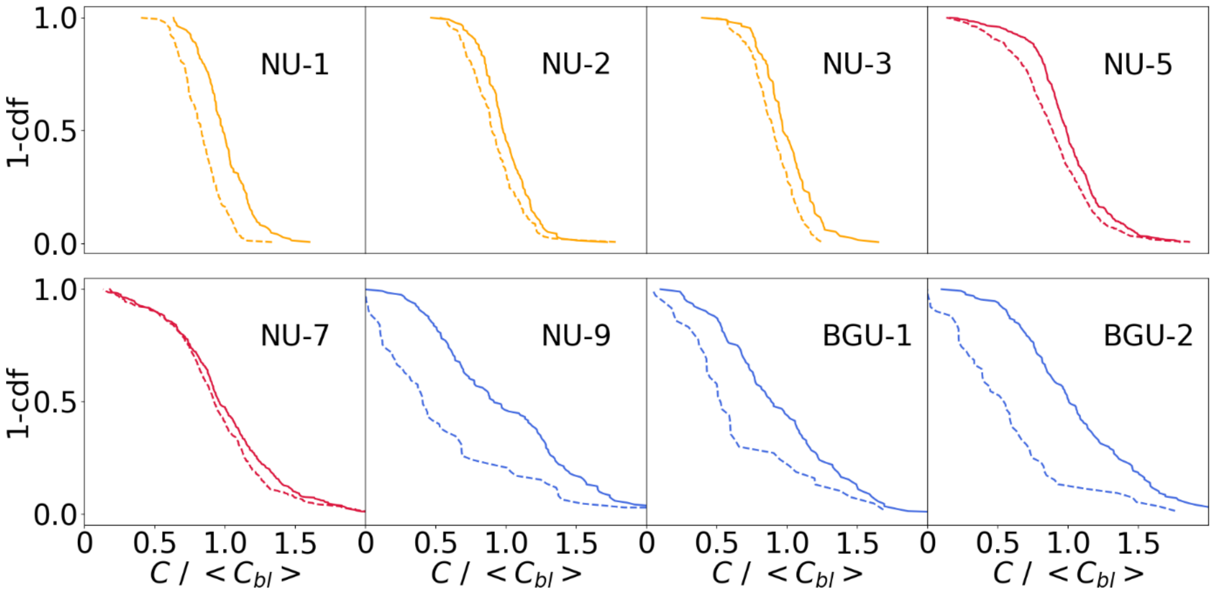


**Supplementary Figure 3:** Celerities for all runs not shown in Figure 2. Experiments show a clear connection between the shear velocity and the subsequent decreases in celerity, with low shear velocity (blue) showing the most pronounced declines, followed by medium (orange) and high (red). The cdfs were taken for the baseline period (solid line) and the last 100 hours of measurement (dashed line).

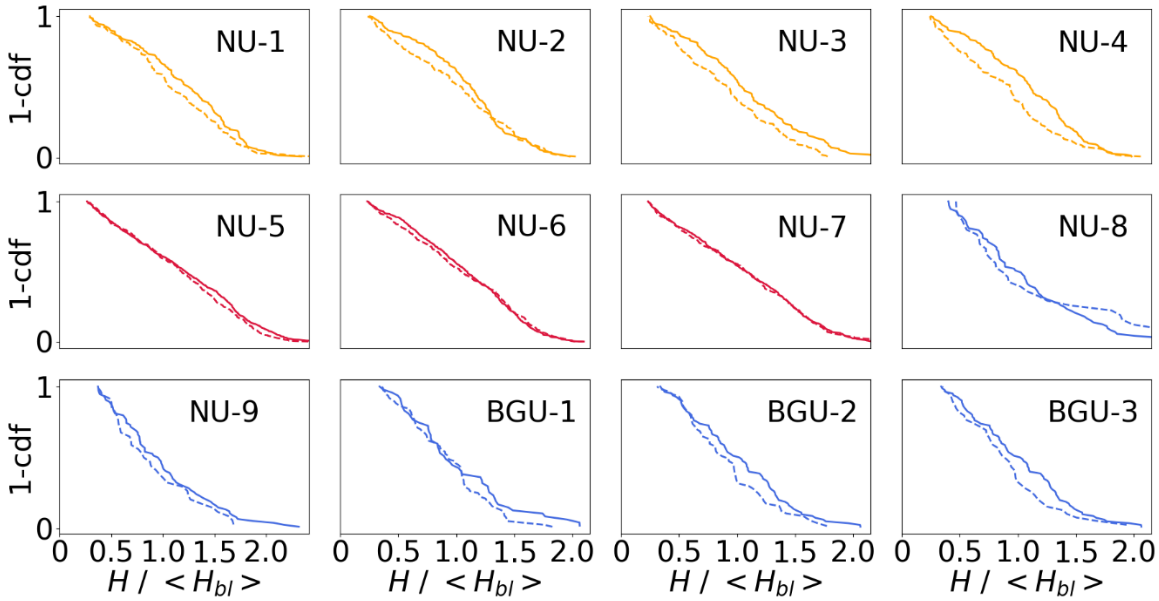


**Supplementary Figure 4:** Bedform height for all runs. The clear relationship seen between decreasing shear velocity and decreasing celerity is less obvious for the height. High shear velocity runs (red) show little evidence of a height change. The medium (yellow) and low (blue) shear velocity runs show a more pronounced drop in height across all experiments. Interestingly, the low shear velocity runs do not show a drop in height beyond what was seen for the medium shear velocity runs.

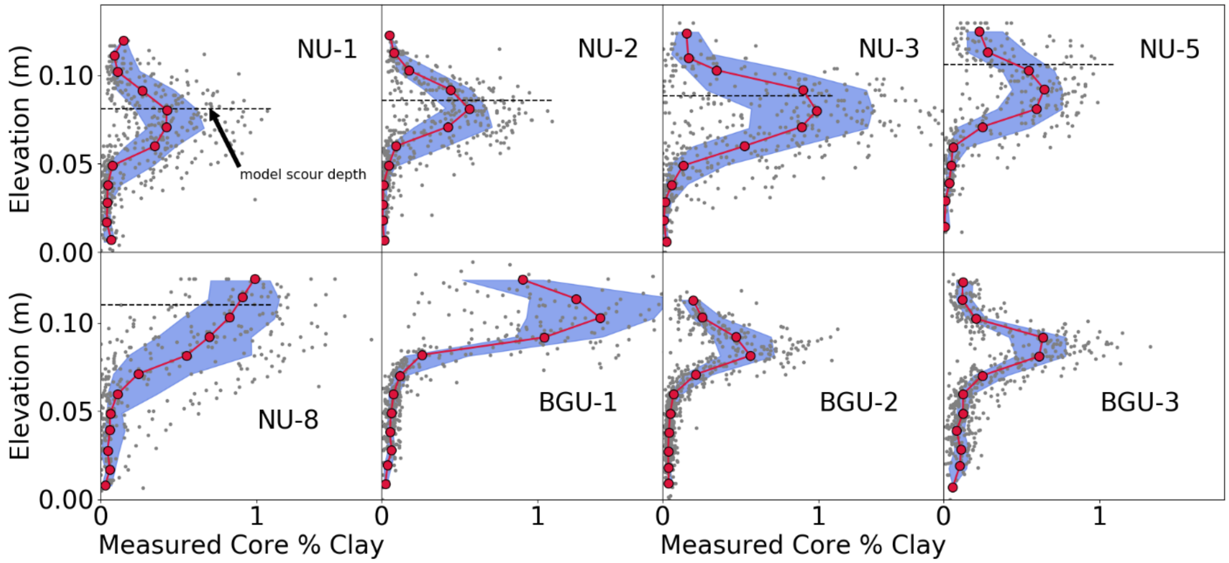


**Supplementary Figure 5:** All available core data (grey dots) for the notated runs. Data scatter is binned every 1 cm and medians are marked with a red dot. The blue shading denotes the interquartile range. The red line connects the medians for ease in visualization. Runs NU-1 to NU-7, BGU-2 and BGU-3 show the formation of a dense subsurface clay stratum underlaying a mainly clay free active layer – the segregated end state. Run NU-8 shows the locked end state, with significant amounts of clay in the active layer halting bed motion. No buried higher concentration clay layer is created; instead clay extends all the way through the active layer. BGU-1 remained in motion but saw the beginning of incipient locking. Significantly more clay is present in the active layer for this run relative to the other BGU runs. The incipient locking started during the final measurement period of this run and was increasing in frequency though did not reach a completely locked state by the end of the experimental run time.

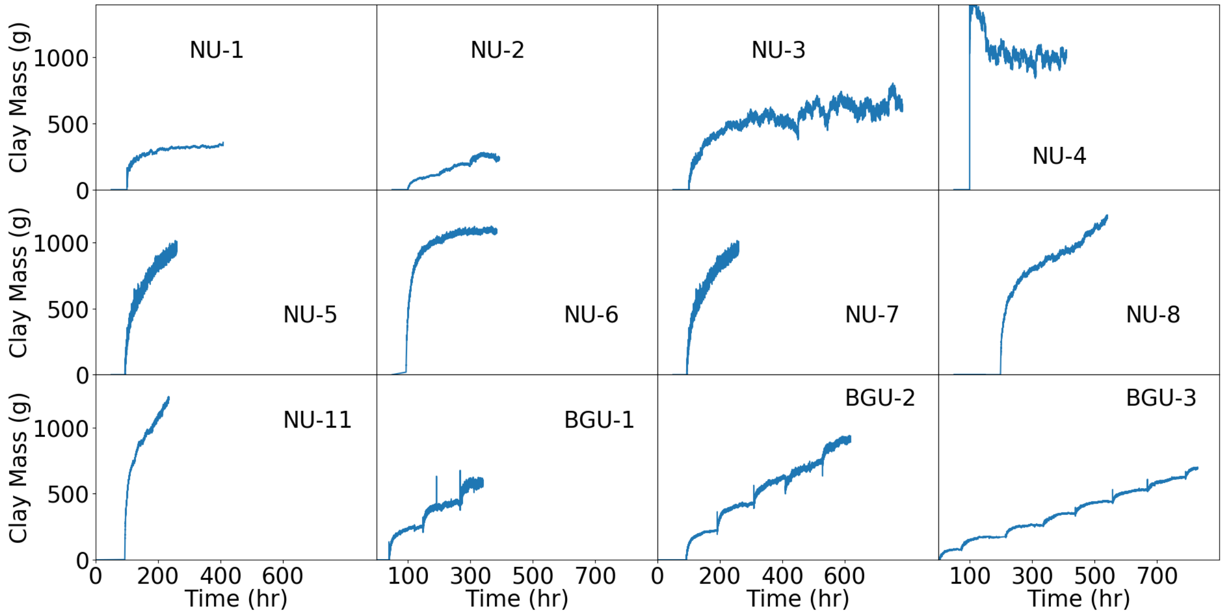


**Supplementary Figure 6:** All available data for the accumulated clay mass in the bed. Clay accumulates with time in all injections.
