# Supplementary material for: Bedform segregation and locking increase storage of natural and synthetic particles in rivers
Source: Nat Commun. 2021 Dec 16;12:7315. doi: 10.1038/s41467-021-27554-4 (PMC8677759; doi:10.1038/s41467-021-27554-4)
Supplement: Supplementary file 2 — Description of Additional Supplementary Files [file 41467_2021_27554_MOESM2_ESM.pdf]

Description of Additional Supplementary Files

File Name: Supplementary Movie 1
Description: From NU-9 illustrating the locking, unlocking, and relocking of initially mobile bedforms. Clay has been in the freestream of the flume 25 minutes (real time) before the video started. As the bedforms are relatively slow, the video has been sped up such that one second of footage equals 7800 seconds of real time to allow for easy visualization. Bedforms move in an erratic fashion before completely locking for an extended period.

File Name: Supplementary Movie 2
Description: Dye injection conducted after NU-7 and sped up such that one second of footage equals just over 300 seconds of real time to allow for easy visualization. All clay was removed from the freestream and blue dye was injected to visualize the propagation of hyporheic flow. Exchange with the mobile sediment region is rapid and dye quickly permeates the active layer. However, dye takes longer to penetrate the low permeability clay layer. Local heterogeneities in the layer allow for flow to penetrate deeper into the flume as does the presence of bedform troughs.
